# Multi-Lineage *BCR-ABL* Expression in Philadelphia Chromosome-Positive Acute Lymphoblastic Leukemia Is Associated With Improved Prognosis but No Specific Molecular Features

**DOI:** 10.3389/fonc.2020.586567

**Published:** 2020-10-23

**Authors:** Satoshi Nishiwaki, Jeong Hui Kim, Masafumi Ito, Matsuyoshi Maeda, Yusuke Okuno, Daisuke Koyama, Yukiyasu Ozawa, Masaharu Gunji, Masahide Osaki, Kunio Kitamura, Yoko Ushijima, Yuichi Ishikawa, Koichi Miyamura, Isamu Sugiura, Hitoshi Kiyoi

**Affiliations:** ^1^ Department of Advanced Medicine, Nagoya University Hospital, Nagoya, Japan; ^2^ Department of Hematology and Oncology, Nagoya University Graduate School of Medicine, Nagoya, Japan; ^3^ Department of Pathology, Japanese Red Cross Nagoya Daiichi Hospital, Nagoya, Japan; ^4^ Division of Pathology, Toyohashi Municipal Hospital, Toyohashi, Japan; ^5^ Medical Genomics Center, Nagoya University Hospital, Nagoya, Japan; ^6^ Division of Hematology and Oncology, Toyohashi Municipal Hospital, Toyohashi, Japan; ^7^ Department of Hematology, Japanese Red Cross Nagoya Daiichi Hospital, Nagoya, Japan; ^8^ Division of Hematology, Ichinomiya Municipal Hospital, Ichinomiya, Japan

**Keywords:** *BCR-ABL*-expressing lineage, multi-lineage, multipotent progenitor, Philadelphia chromosome-positive acute lymphoblastic leukemia, uni-lineage

## Abstract

**Background:**

Recently, various blood cell lineages expressing the *BCR-ABL* fusion gene in Philadelphia chromosome (Ph)-positive acute lymphoblastic leukemia (ALL) have been reported. However, the biological and clinical significance of these *BCR-ABL* lineages has not been established; therefore, we aimed to clarify the impacts of these different *BCR-ABL*-expressing lineages.

**Patients:**

Multi-lineage *BCR-ABL* expression (multi-Ph) was defined as *BCR-ABL* expression outside of the B-lineage compartment, as determined by fluorescence *in situ* hybridization (FISH) in peripheral blood neutrophils and bone marrow clots, and flow cytometry-sorted polymerase chain reaction (PCR). We analyzed *IKZF1* deletion patterns by PCR, examined gene expression profiles using RNA sequencing, and compared treatment outcomes across different *BCR-ABL*-expressing lineages.

**Results:**

Among the 21 multi-Ph patients in our 59-patient cohort (36%), *BCR-ABL* expression was detected at the multipotential progenitor level. However, no *IKZF1* deletion patterns or gene expression profiles were identified that were specific for multi-Ph. However, multi-Ph patients were found to have better survival rates than patients with uni-lineage *BCR-ABL* expression [event-free survival (EFS): 74 vs. 33%, *P* = 0.01; overall survival (OS): 79 vs. 44% at 4 years, *P* = 0.01]. In multivariate analyses, multi-Ph was identified as a good prognostic factor for both EFS and OS.

**Conclusion:**

We confirmed that more than one-third of Ph+ALL patients could be classified as mutli-Ph. Although no specific molecular characteristics were identified for multi-Ph, this phenotype was associated with better treatment outcomes.

## Introduction

Cells that express *BCR-ABL* outside of the B-lineage compartment have been reported in a proportion of patients with Philadelphia chromosome-positive (Ph+) B-precursor acute lymphoblastic leukemia (ALL) (1–4). In most cases, the fusion gene *BCR-ABL*, which is the genetic abnormality associated with Ph+ALL, is expressed only in B-lymphocytes; however, in some cases, *BCR-ABL* was also expressed in the myeloid lineage, suggesting that these cells originated from a separate differentiation stage than B-lineage cells.

The expression of *BCR-ABL* by non-lymphocytic lineages is a relatively recent finding, and the biological and clinical significance of this finding has yet to be clarified. The differentiation stage during which the genetic abnormality occurs must be identified, and whether leukemic cells originating from various differentiation stages are molecularly heterogeneous must be determined. Furthermore, whether similar genetic abnormalities with different origins have varying influences on patient prognosis must also be determined. The occurrence of genetic abnormalities in cells other than leukemic cells may also affect the detection of minimal residual disease (MRD) (3, 4), which is currently crucial for determining an optimal treatment strategy for Ph+ALL (4–7). In this study, we confirmed two types of adult Ph+ALL that derive from different leukemic origins. In addition, we analyzed the genetic characteristics and therapeutic responses of Ph+ALL associated with genetic abnormalities in cells other than leukemic blast cells.

## Materials and Methods

### Patients

Fifty-nine adult Ph+ALL patients were deemed eligible for this retrospective study. Patients with *de novo* Ph+ALL were included if peripheral blood or bone marrow samples were available. No patients had any history of chronic myeloid leukemia (CML). Ph+ALL was confirmed through the detection of the Ph chromosome by fluorescence *in situ* hybridization (FISH) analysis and the positive detection of *BCR-ABL* fusion transcript expression by reverse-transcription-polymerase chain reaction (RT-PCR) analysis. The immunophenotyping of leukemic blast cells was performed using flow cytometry or immunohistochemistry, at individual hospitals (7). The copy numbers of *BCR-ABL* transcripts identified in each sample were normalized against the expression levels of *GAPDH*. The threshold for quantification was 50 copies/µg RNA, which corresponded to a minimal sensitivity of 10^−5^ (8). In the present study, the inability to detect *BCR-ABL* by RT-PCR was defined as complete molecular remission (CMR). All patients were treated with tyrosine kinase inhibitor (TKI) ± conventional chemotherapy. No criteria were established for TKI selection or the performance of allogeneic hematopoietic cell transplantation (allo-HCT). Treatment strategies depended on each physician’s judgments and the treatment policy of each hospital. Representative treatments were based on studies performed by the Japan Adult Leukemia Study Group for Ph+ALL [Ph+ALL202 (9), Ph+ALL208 (10), or Ph+ALL213 (11)], and written informed consent was obtained from each patient upon treatment and sample collection. Each hospital’s institutional review board approved the study.

### Fluorescence *In Situ* Hybridization of the *BCR-ABL* Fusion Gene

To assess the expression of the *BCR-ABL* fusion gene outside of leukemic blast cells, we performed FISH analyses on two different specimens: peripheral blood (PB) neutrophils (PN-FISH) (12) and bone marrow clot sections (BM-FISH) (13). A Vysis LSI *BCR/ABL* ES Dual-Color Translocation Probe (Abbott Laboratories, Abbott Park, IL, USA) was hybridized with PB and BM samples (2, 14). The denaturation of the chromosome/probe, hybridization, and washing were performed according to the manufacturer’s instructions. Multi-Ph was defined as the detection of *BCR-ABL* fusion gene signals in segmented neutrophils, including of PB mononuclear cells and PB neutrophils. Similarly, the detection of *BCR-ABL* fusion gene signals in myeloperoxidase-positive cells and polynuclear megakaryocytes in 10% formalin-fixed paraffin-embedded bone marrow clot sections by BM-FISH was defined as multi-Ph.

### Flow Cytometry and Cell Sorting

The expression of *BCR-ABL* was examined by dividing bone marrow/peripheral blood cells into the following fractions, as defined in previous studies (1, 15): CD34^+^CD38^–^CD19^–^CD3^–^ [hematopoietic stem cells (HSCs) and multipotent progenitor (MPP) cells], CD34^+^CD38^+^CD19^–^CD3^–^ [myeloid and lymphoid progenitors (Pro)], CD34^+^CD19^+^CD20^–^CD3^–^ [leukemia cells without CD20 coexpression; leukemia associated immunophenotype (LAIP) 20^–^], CD34^+^CD19^+^CD20^+^CD3^–^ (leukemia cells with CD20 coexpression; LAIP 20^+^), CD34^–^CD19^+^CD20^+^ (mature B-cells), CD34^–^CD19^–^CD20^–^CD3^+^ (mature T-cells), CD34^+^CD19^–^CD13/33^+^CD10^–^CD16^–^ (early myeloid compartment), CD34^–^CD19^–^CD13/33^+^CD10^–^CD16^–^ (late myeloid compartment), and CD34^–^CD19^–^CD13/33^+^CD10^–^CD16^+^ (mature myeloid compartment). Fluorescence-activated cell sorting was performed using a FACS Aria II flow cytometer (BD Biosciences, San Jose, CA, USA), applying 4’,6-diamidino-2-phenylindole (DAPI) for live/dead cell discrimination. All antibodies were purchased from BD, Beckman Coulter (Indianapolis, IN, USA) or Biolegend (San Diego, CA, USA). Fluorochrome-conjugated antibodies included CD19 (J3-119), CD20 (2H7), CD34 (8G12), CD38 (HB7), CD10 (HI10a), CD3 (2H7), CD16 (3G8), CD13 (SJ1D1), and CD33 (P67-6) antibodies.

### RT-PCR Analysis

Total RNA was extracted from sorted cells, using an RNeasy MiniKit or a QIAamp RNA Blood Mini Kit (QIAGEN, Hilden, Germany), and reverse transcribed, using the SuperScript II Reverse Transcriptase Kit (Thermo Fisher Scientific, Waltham, MA, USA), according to the manufacturer’s instructions. *BCR-ABL* transcript expression levels were quantitated based on a real-time fluorescence detection method using an ABI Prism7300 Sequence Detection System and Taqman^®^ Gene Expression Assay probe (Applied Biosystems, Foster City, CA, USA). *GAPDH* served as a control for cDNA quality. To detect *IKZF1* isoforms, *IKZF1* transcripts were amplified from cDNA, as previously described (16). *BCR-ABL* fusion transcripts that were detected outside of the lymphoid compartment, using a flow cytometry-sorted PCR method (FCM-PCR), were classified as multi-Ph.

### RNA Sequencing

All cryopreserved samples from which high-quality RNA could be extracted were submitted to RNA sequencing. We prepared non-directional sequencing libraries, using a NEBNext Ultra II RNA Prep Kit for Illumina and a NEBNext Poly(A) mRNA Magnetic Isolation Module (New England Biolabs, Ipswich, MA, USA), according to the manufacturer’s instructions. We ran the prepared libraries using a HiSeq 2500 next-generation sequencing platform (Illumina, San Diego, CA, USA) to obtain an average of 100 million reads per sample. The obtained reads were aligned against the hg19 reference genome, using TopHat2 (17), and fragments per kilobase of exon per million mapped reads (FPKM) values were calculated using Cufflinks (18). For clustering analysis, we used HTSeq (19) for raw read counting and DESeq2 (20) for variance stabilizing transformation. Cluster 3.0 (21) was used to filter genes with default parameters. Finally, we used pyclust (22) to perform average linkage hierarchical clustering using Pearson’s correlation coefficient. Bootstrapping was performed for 1,000 permutations to calculate the bootstrap probability and to obtain approximately unbiased values.

### Survival Analysis

Primarily, we analyzed event-free survival (EFS), from the date of diagnosis to the date of any event, including death by any cause, failure to achieve remission after induction therapy, relapse in any site, and second malignancy (23–25); for patients without an event, the date of the last visit was used. EFS probabilities were estimated using the Kaplan–Meier method, and *P-*values were calculated using a log-rank test (26, 27). We also analyzed overall survival (OS), from the date of diagnosis to the date of death or the date of the last visit. Univariate and multivariate analyses were performed using a Cox proportional hazard regression model (28). A backward stepwise procedure was used to develop a final model, based on a *P*-value threshold of 0.05. The covariates considered were patient sex (male vs. female), patient age (<65 y vs. ≥65 y), white blood cell count at diagnosis (< 30,000/µl vs. ≥ 30,000/µl), *BCR-ABL* transcript [(e1a2 vs. b2a2 or b3a2 (b2a2/b3a2)], additional cytogenetic abnormality (no vs. yes), type of TKI (imatinib vs. dasatinib), allo-HCT (no vs. yes) and *BCR-ABL* lineage (uni-lineage vs. multi-lineage). A significance level of *P <*0.05 was used for all analyses.

## Results

### Expression of *BCR-ABL* Outside of Leukemic Blast Cells

**Figure 1:**
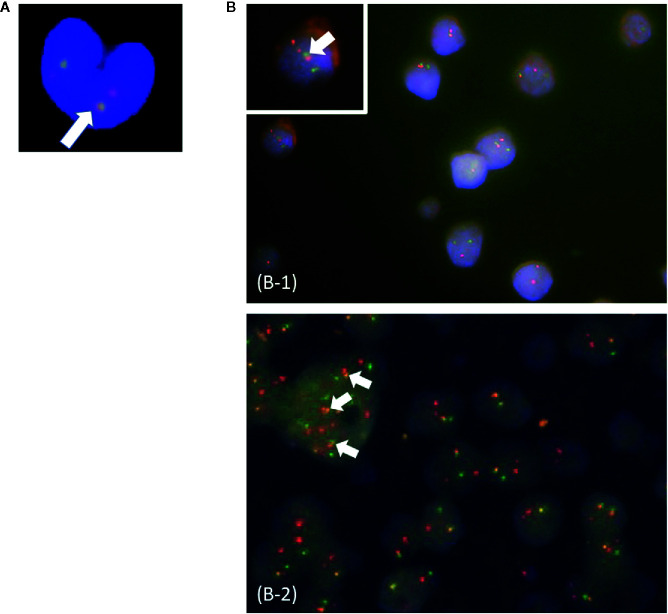
Fluorescence *in situ* hybridization (FISH) for the *BCR-ABL* fusion gene. **(A)** Peripheral blood neutrophil-FISH (PN-FISH); **(B)** FISH in bone marrow clot sections (BM-FISH). (B-1) shows *MPO*-positive myeloid cells. (B-2) shows a megakaryocyte. White arrows indicate *BCR-ABL* fusion signals. Red signals are from *ABL* probes, and green signals are from *BCR* probes.

**Figure 2:**
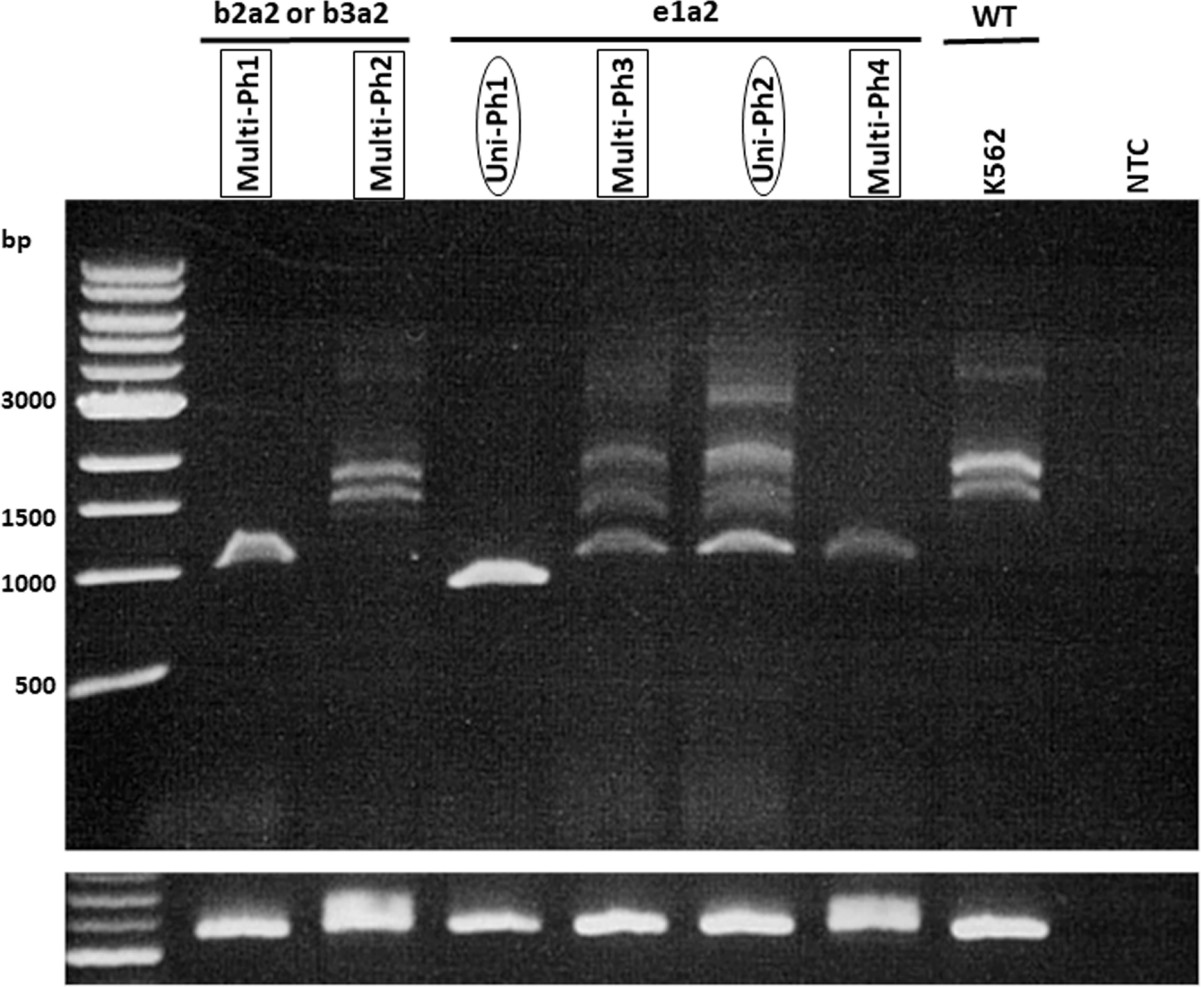
RT-PCR for *IKZF1* transcripts. RT–PCR for *IKZF1* transcripts (using exon 0- and 7-specific primers) in both uni-lineage Ph and multi-lineage Ph cases. K562 cell lines were used as the *IKZF1* wild-type control.

In this study, patients were classified as multi-Ph through at least one of three approaches (PN-FISH, BM-FISH, and FCM-PCR). All other patients were defined as uni-lineage Ph leukemia (uni-Ph). PN-FISH was performed for 19 patients (**Figure 1A**), BM-FISH was performed for 49 patients (**Figure 1B**), and FCM-PCR was performed for six patients. A total of 21 patients (36%) were identified as multi-Ph; a discrepancy between detection methods was observed in only one patient (PN-FISH: uni-Ph, BM-FISH: multi-Ph). In two patients, *BCR-ABL* signals were also detected in megakaryocytes (**Figure 1B-2**). Four multi-Ph individuals expressed e1a2, whereas the remaining 17 individuals with multi-Ph expressed b2a2/b3a2. The frequency of multi-Ph was significantly higher among patients expressing b2a2/b3a2 than those expressing e1a2 (81 vs. 11%, *P* < 0.001, **Table 1**).

**Table 1:** Characteristics of patients with Ph+ALL according to *BCR-ABL-*expression lineage.

	Uni-lineage	(%)	Multi-lineage	(%)	*P*
No. of patients	38		21		
Sex					0.08
Male	20	53	6	29	
Female	18	47	15	71	
Age					0.51
<65 y	22	58	14	67	
≥65 y	16	42	7	33	
WBC at diagnosis					0.06
<30,000/µl	24	63	8	38	
≥30,000/µl	14	37	13	62	
*BCR-ABL* transcript					<0.001
e1a2	34	89	4	19	
b2a2 or b3a2	4	11	17	81	
Additional cytogenetic abnormality					0.56
(−)	12	32	9	43	
(+)	22	58	12	57	
Unknown	4	11	0	0	
Tyrosine kinase inhibitor					0.02
Imatinib	26	68	8	38	
Dasatinib	10	26	12	57	
Other	2	5	1	5	
Allogeneic hematopoietic cell transplantation					0.13
(−)	24	63	9	43	
(+)	14	37	12	57	

### Fraction Expressing *BCR-ABL* and *IKZF1* Deletion

FCM-PCR was evaluated in six patients (4 with e1a2 and 2 with b2a2/b3a2). *BCR-ABL* fusion transcripts were detected in the LAIP 20^–^ and LAIP 20+ compartments, as expected, but the HSCs/MPP and Pro compartments were also *BCR-ABL*-positive in four patients (2 with e1a2 and 2 with b2a2/b3a2).

*IKZF1* deletion was detected in five of six evaluated patients (4 with e1a2 and 1 with b2a2/b3a2). No deletion patterns were found associated with multi-Ph (**Figure 2**). In three patients, *IKZF1* deletion was detected in the HSCs/MPP and Pro compartments, as well as in the LAIP 20^–^ and LAIP 20^+^ compartments, which was consistent with the findings for *BCR-ABL*-positive compartments.

### Gene Expression According to Ph Lineage

**Figure 3:**
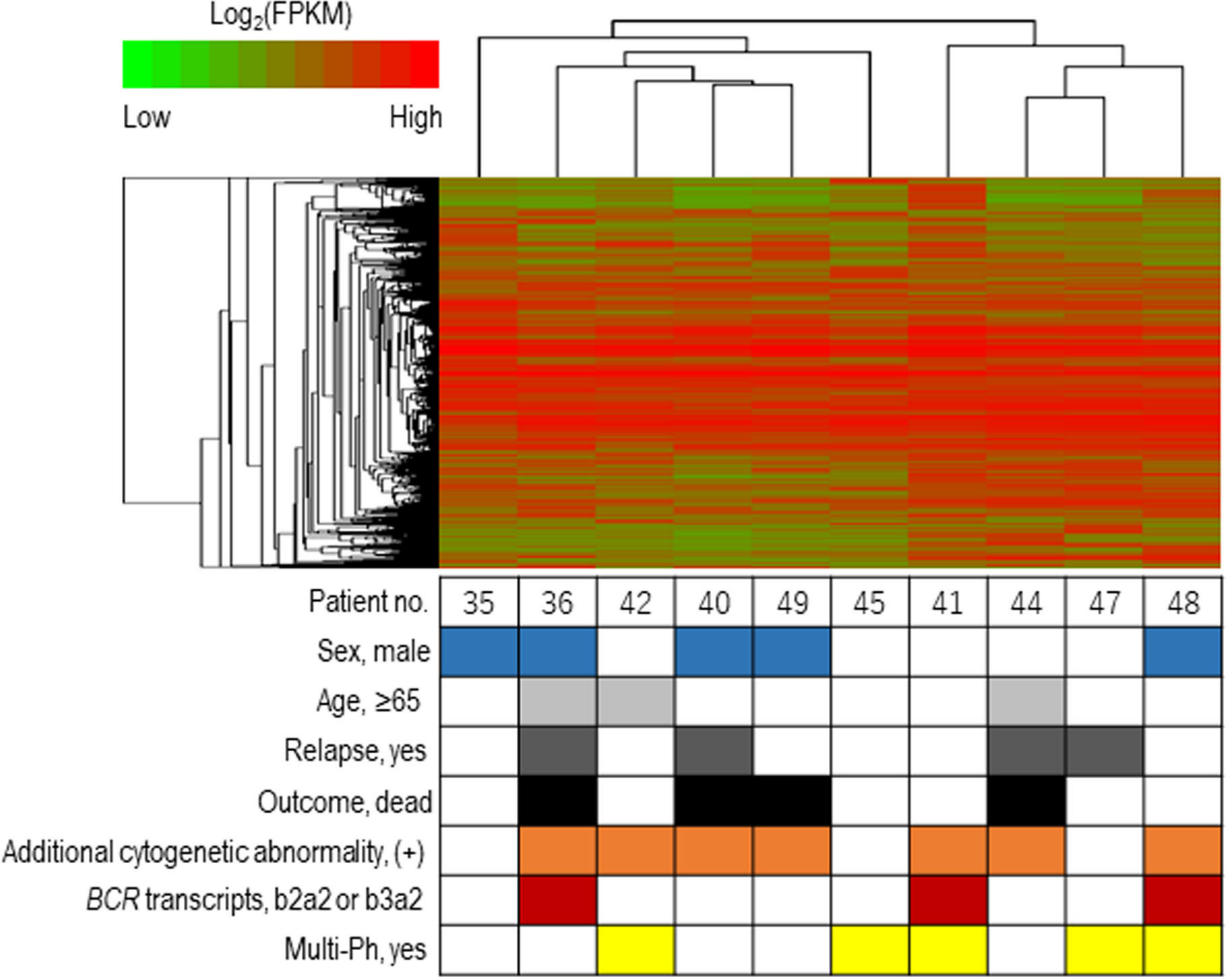
Expression profile-based clustering. Hierarchical clustering was performed based on the expression profiles obtained by RNA sequencing. The heat map shows the hierarchically clustered samples, with Pearson’s correlations, according to the significantly differentially expressed genes. Each column indicates an individual patient, and the lines at the top indicate the cluster dendrogram grouping of patients based on their similarities.

The expression profiles of leukemic blast cells were assessed by performing a hierarchical clustering analysis, with bootstrapping, in ten patients (uni-Ph: 5, multi-Ph: 5). Blast cells derived from patients with multi-Ph were not grouped into a cluster that was distinguishable from those derived from patients with uni-Ph, suggesting that the global expression profiles of uni-Ph and multi-Ph blast cells were similar (**Figure 3**, **Supplementary Table**).

### Treatment Response According to Ph Lineage

**Figure 4:**
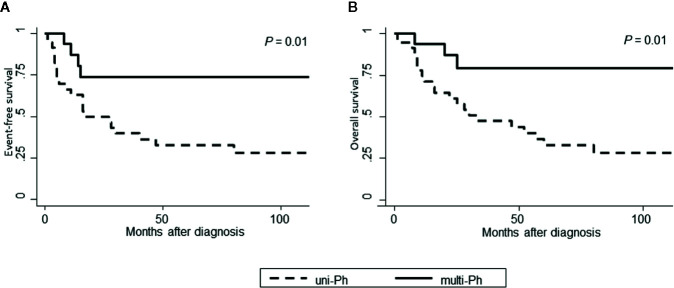
Patient outcomes, according to *BCR-ABL*-expressing lineage. **(A)** Event-free survival; **(B)** Overall survival.

Both EFS and OS were significantly higher in patients with multi-Ph than in patients with uni-Ph (EFS: 74 vs. 33%, *P* = 0.01; OS: 79 vs. 44% at 4 years, *P* = 0.01, **Figure 4**). However, the CMR rate following the first induction therapy was significantly lower in patients with multi-Ph than in those with uni-Ph (11 vs. 56%, *P* = 0.003). In two patients with multi-Ph, BM-FISH at complete hematological remission showed positive *BCR-ABL* signals only outside of leukemic blast cells. In multivariate analyses, both *BCR-ABL* lineage and age were identified as significant prognostic factors for both EFS and OS (**Table 2**). Neither the type of TKI nor allo-HCT were found to be significant prognostic factors.

**Table 2:** Univariate and multivariate analyses of factors influencing survival among patients with Ph+ALL.

Covariates	Univariate			Multivariate		
	HR	95%CI	*P*	HR	95%CI	*P*
Event-free survival						
Sex						
Male	1.00					
Female	0.99	(0.45–2.16)	0.98			
Age						
<65 y	1.00			1.00		
≥65 y	3.41	(1.57–7.45)	0.002	4.26	(1.91–9.48)	<0.001
WBC at diagnosis						
<30,000/µl	1.00					
≥30,000/µl	1.41	(0.64–3.08)	0.40			
*BCR-ABL* transcript						
e1a2	1.00					
b2a2 or b3a2	0.41	(0.15–1.08)	0.07			
Additional cytogenetic abnormality						
(−)	1.00					
(+)	1.31	(0.56–3.06)	0.54			
Tyrosine kinase inhibitor						
Imatinib	1.00					
Dasatinib	0.44	(0.18–1.08)	0.07			
Allogeneic hematopoietic cell transplantation						
(−)	1.00					
(+)	0.25	(0.10–0.60)	0.002			
*BCR-ABL* lineage						
Uni-lineage	1.00			1.00		
Multi-lineage	0.27	(0.09–0.79)	0.02	0.22	(0.07–0.63)	0.005
Overall survival						
Sex						
Male	1.00					
Female	0.88	(0.39–1.98)	0.76			
Age						
<65 y	1.00			1.00		
≥65 y	3.69	(1.63–8.36)	0.002	4.61	(2.00–10.6)	<0.001
WBC at diagnosis						
<30,000/µl	1.00					
≥30,000/µl	1.65	(0.73–3.70)	0.23			
*BCR-ABL* transcript						
e1a2	1.00					
b2a2 or b3a2	0.48	(0.18–1.28)	0.14			
Additional cytogenetic abnormality						
(−)	1.00					
(+)	1.46	(0.60–3.59)	0.41			
Tyrosine kinase inhibitor						
Imatinib	1.00					
Dasatinib	0.44	(0.17–1.14)	0.09			
Allogeneic hematopoietic cell transplantation						
(−)	1.00					
(+)	0.30	(0.12–0.72)	0.007			
*BCR-ABL* lineage						
Uni-lineage	1.00			1.00		
Multi-lineage	0.25	(0.07–0.83)	0.02	0.19	(0.06–0.65)	0.008

## Discussion

In this study, we used multiple approaches to confirm the heterogeneity of adult Ph+ALL according to *BCR-ABL* expression lineages (1, 2). *BCR-ABL* signals were observed outside of leukemic blast cells in 36% of patients with Ph+ALL. We detected *BCR-ABL* signals not only in myeloid cells but also in megakaryocytes, which suggested the involvement of early differentiation stages in multi-Ph, including those that occur close to HSCs (29).

Three techniques were used to identify multi-Ph in this study: PN-FISH, BM-FISH, and FCM-PCR. Therefore, even in cases for which multi-Ph was difficult to determine using one method, the complementary use of multiple approaches allowed for the identification of multi-Ph. Most cells were classified as leukemic blast cells at diagnosis, and morphological judgments alone were not sufficient for the identification of fractions other than leukemic blast cells. Myeloperoxidase double staining was effective in such cases. Although FCM-PCR is a sensitive method, the number of cases we analyzed remained small because cryopreserved cells tended to feature too many dead cells to obtain an adequate signal.

To clarify the biological differences between uni-Ph and multi-Ph, we analyzed the deletion pattern of *IKZF1*, the most frequent genetic abnormality associated with Ph+ALL (16). However, no distinct *IKZF1* deletion patterns were associated with multi-Ph. Gene expression analysis also failed to identify any clusters specific to multi-Ph. In addition to the limited number of samples included in this analysis, the lack of differences in gene expression may be associated with the use of stored samples, which were obtained at diagnosis and primarily contained leukemic blast cells. Because the only difference between uni-Ph and multi-Ph is the process through which the patient develops leukemia, any differences in the expression profile may disappear once a cell differentiates into a leukemia blast cell. Therefore, the analysis of other cell types or different differentiation stages between uni-Ph and multi-Ph may be necessary to observe genetic phenotypes.

Survival rates were significantly different between patients with uni-Ph and those with multi-Ph, consistent with a previous small-scale study (2). The better survival rate observed for patients with multi-Ph may be due to differences in the cell differentiation stage affected by TKI treatment between the two lineages, instead of specific genetic differences in leukemic cells. Leukemic blast cells are thought to derive from short-term HSC (ST-HSC) or the MPP level of development in multi-Ph, whereas they derive from common lymphoid progenitors (CLP)/lymphoid-primed multipotent progenitors (LMPP) in uni-Ph (30, 31). TKI treatment, alone, demonstrated a weak effect on the elimination of quiescent ST-HSCs/MPPs; however, when combined with chemotherapy, TKI may activate quiescent HSCs (32), resulting in the better eliminating of leukemic stem cells (33). In contrast, uni-Ph ALL, which is thought to originate from CLPs/LMPPs, includes ALL derived from aggressive B-1 progenitor cells, which is associated with poor TKI treatment outcomes (34, 35).

This study has several limitations. First, the number of samples that could be analyzed was limited. Because all available samples were used, the selection was not arbitrary; however, a limited selection of samples was available for data collection, such as few samples from remission cases. In addition, the MRD data were only based on PCR for *BCR-ABL*, and no MRD data associated with Immunoglobulin (Ig)/T-cell receptor (TCR) gene rearrangements were collected in this study. In Japan, Ig/TCR analysis is not commonly performed to detect MRD in adult Ph+ALL patients. Discrepancies in MRD data between *BCR-ABL* expression and Ig/TCR analyses can indicate the presence of *BCR-ABL* expression outside of lymphoblasts (3, 4). The histopathological distinction between blast cells and other blood cells using tissue FISH could complement the lack of Ig/TCR data by detecting the expression of *BCR-ABL* in cell types other than leukemic blast cells.

In conclusion, we confirmed *BCR-ABL* expression outside of leukemic blast cells in more than one-third of the Ph+ALL patients examined in this study. Although unique genetic patterns could not be identified, these patients revealed good prognoses following treatment, including TKI treatment.

## Data Availability Statement

All datasets generated for this study are included in the article/**Supplementary Material**. Further inquiries can be directed to the corresponding author.

## Ethics Statement

The studies involving human participants were reviewed and approved by Institutional review board of Nagoya University. The patients/participants provided their written informed consent to participate in this study.

## Author Contributions

SN, IS, and HK designed the research and interpreted the data. JHK, YU, and YI performed flow cytometric and PCR assays. MI, MM, and MG performed immunohistochemical assays. YOk performed RNA sequencing and bioinformatic analyses of the sequencing data. DK, YOz, MO, KK, and KM collected specimens and provide the data of patients. SN performed statistical analyses, and wrote the manuscript. All authors contributed to the article and approved the submitted version.

## Funding

This study was supported in part by JSPS KAKENHI Grant Number JP17K16186 and JP20K08730.

## Conflict of Interest

HK received research funding from Chugai Pharmaceutical Co., Ltd., Kyowa Hakko Kirin Co., Ltd., Zenyaku Kogyo Co., Ltd., FUJIFILM Corporation, Daiichi Sankyo Co., Ltd., Astellas Pharma Inc., Otsuka Pharmaceutical Co., Ltd., Nippon Shinyaku Co., Ltd., Eisai Co., Ltd., Pfizer Japan Inc., Takeda Pharmaceutical Co., Ltd., Novartis Pharma K.K., Sumitomo Dainippon Pharma Co., Ltd., Sanofi K.K., and Celgene Corporation, consulting fees from Astellas Pharma Inc., Amgen Astellas Bio Pharma K.K., and Daiichi Sankyo Co., Ltd., and honoraria from Bristol-Myers Squibb, Astellas Pharma Inc., and Novartis Pharma K.K. These companies are not directly involved in any part of this study.

The remaining authors declare that the research was conducted in the absence of any commercial or financial relationships that could be construed as a potential conflict of interest.
